# Immersion ultrasound improves the repeatability of shear-wave elastography for measuring median nerve elasticity

**DOI:** 10.1186/s13018-023-04097-6

**Published:** 2023-08-23

**Authors:** Zhijun Zhang, Hui Wang, Xiaoya Ding, Shu He

**Affiliations:** 1 Department of Ultrasound, University-Town Hospital of Chongqing Medical University, Chongqing, 401331 China; 2 Medical Affairs Department, The Fifth People’s Hospital of Chongqing, Chongqing, 400030 China

**Keywords:** Shear wave elastography, Median nerve, Repeatability

## Abstract

**Objective:**

To study the factors that influence the measurement of median nerve elasticity, to provide a more reproducible test for the assessment of median nerve elasticity using shear wave elasticity (SWE), and to reduce operator empirical dependence. To compare the repeatability of the median nerve elasticity measurement using immersion SWE with that using contact SWE, analyze the factors affecting SWE measurement, and provide a more repeatable method for doctors without SWE operation experience.

**Methods:**

Two doctors without SWE operation experience measured the median nerve mean elastic modulus (Emean) at the same position and at different time points on the right wrist of 58 healthy volunteers using immersion and contact ultrasound methods. The intraobserver and interobserver repeatability of measurements was assessed using the interclass correlation coefficient (ICC), while the repeatability was assessed using the Bland–Altman diagram.

**Results:**

The intraobserver and interobserver repeatability of the median nerve elasticity measured via contact SWE by inexperienced operators were classified as good, with ICCs of 0.633 (95% CI 0.380–0.783) and 0.552 (95% CI 0.243–0.735), respectively. The intraobserver and interobserver repeatability of the median nerve elasticity measured by immersion SWE were very good, with ICCs of 0.975 (95% CI 0.958–0.985) and 0.942 (95% CI 0.902–0.966), respectively. The intraobserver and interobserver Bland–Altman diagram of median nerve elasticity measured by immersion SWE showed that 98% of the points fell within the 95% limits of agreement. The intraobserver and interobserver Bland–Altman diagram of median nerve elasticity measured by contact SWE showed that 94% of the points fell within the 95% limits of agreement.

**Conclusion:**

Immersion ultrasound can improve the repeatability of median nerve elasticity measurements by inexperienced operators.

## Introduction

Shear wave elasticity (SWE) has been widely used in clinical practice, and it has been proven to have an important reference value in judging benign and malignant tumors and musculoskeletal lesions [1, 2]. In recent years, research using SWE to evaluate neuropathy has also progressed. Some studies have shown that nerves generally do not have morphological changes in the early stage of lesions, but fibrotic changes may occur, resulting in increased nerve stiffness [3, 4] and allowing the early detection of neuropathy for early intervention. Assessing the change in median nerve stiffness is helpful for diagnosing carpal tunnel syndrome (CTS) [5]. The median nerve was stiffer in patients with CTS than in normal people. Median nerve stiffness in patients with CTS of different severities is also different. The median nerve was significantly stiffer in moderate and severe patients than in mild patients [6–8]. Ultrasound elastography has the potential to identify CTS of varying severity [7]. Although SWE has certain clinical significance in the detection of CTS of the median nerve, performing measurements using SWE requires the operator to have a certain level of experience, skill and technique to obtain high repeatability. Moreover, it is difficult to avoid the pressure of the ultrasound probe on the median nerve during the measurement process, which will increase the pre-stiffness of the median nerve and likely to affect the accuracy and repeatability of the measurement. In this study, researchers proposed a new measurement method with the aims of avoiding the pressure effect of the ultrasound probe on the median nerve, reducing the difficulty and experience dependence of the operation, and providing a better method for measuring the elasticity of the median nerve for doctors without SWE experience.

## Materials and methods

### Statement of ethics

This study was approved by the Ethics Committee of the University-Town Hospital of Chongqing Medical University (LL-202264), and the subjects’ written informed consent was obtained. All methods were performed in accordance with the Declaration of Helsinki.

### Research objects

Fifty-eight healthy volunteers recruited from August to December 2021 were selected. Exclusion criteria: history of forearm surgery, skin scars, diabetes, peripheral neuropathy and other factors that may induce a change in median nerve stiffness.

### Instrument and equipment selection

The equipment used was the French Sonic SuperSonic Imagine AixPlorer ultrasonic diagnostic instrument and the 4–15 MHz linear array probe. The immersion SWE equipment uses a rectangular transparent plastic water tank with a length of 80 cm, a width of 30 cm and a height of 20 cm, and tap water serves as the ultrasonic coupling medium.

### Measurement method

#### Contact SWE measurement method

**Fig. 1:**
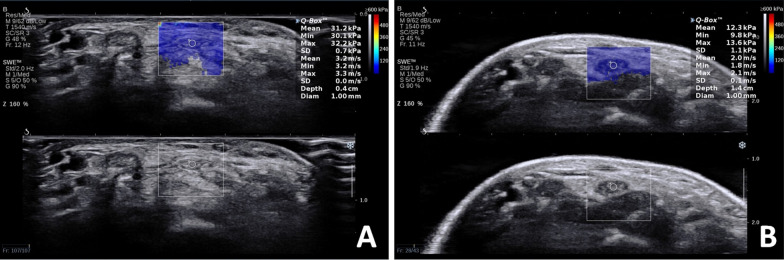
Two methods of SWE display median nerve images. *Note*: **A** Contact SWE shows the median nerve. **B** immersion SWE shows the median nerve

Fifty-eight volunteers sat upright, with their right forearm naturally relaxed and placed on the examination table. Their fingers were naturally bent, and a line was drawn at the level of the pisiform bone of the right hand. This line was drawn on the palm side of the forearm, parallel to the skin crease of the wrist. The examiners were inexperienced in SWE but were not inexperienced in musculoskeletal ultrasound. The two medical examiners have been engaged in ultrasound diagnosis for 1 year and able to identify the ultrasound images of the median nerve but with no practical experience in SWE. Two examiners were recruited for this study and trained on the SWE operation procedure for 30 min before the experiment so that the examiners could complete the operation independently. At different time points, the examiners applied ultrasonic coupling agent to the right wrist of 58 healthy volunteers, then scanned the median nerve in cross section, adjusted the image, started SWE, and after the image was stabilized, captured and measured the Emean of median Nerve; the Q-Box diameter was 1 mm (Fig. 1). The average value of two consecutive measurements obtained by examiner 1 was the first measurement. One hour later, examiner 1 repeated the above measurement method to obtain their second average measurement. Examiner 2 obtained the first and second measurement results according to the above-mentioned measurement method in the same sequence, leading to 4 sets of SWE measurement data using contact SWE. All examiners were blinded to the measurement results.

#### Immersion SWE measurement method

**Fig. 2:**
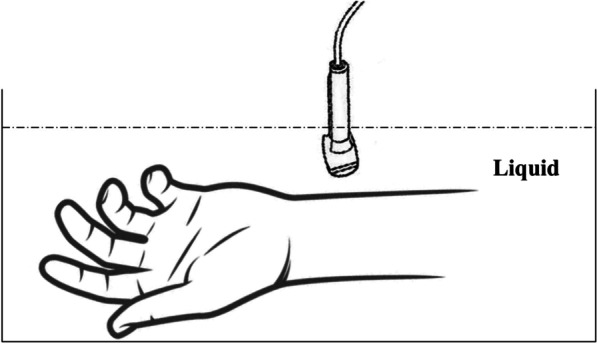
Schematic diagram of median nerve measurement by immersion SWE

Room-temperature tap water was poured into the sink, and the surface of the water exceeded the skin at the wrist by approximately 2 cm. The probe was placed in a protective sheath and immersed in the water to perform the examination, with the probe kept 1 cm away from the skin (Fig. 2). Two examiners at different time points and 58 volunteers were subjected to immersion SWE to measure the Emean of the median nerve at the marker. The specific measurement steps of the immersion SWE are the same as those of the above-mentioned contact ultrasonic method, and 4 groups of measurement data values of the immersion method were obtained.

### Definition of repeatability

The intraobserver repeatability was defined as the intraclass correlation coefficient (ICC) measured twice at different time points by the same examiner using the same method. Interobserver repeatability was defined as ICC measured by two examiners using the same method. Repeatability is divided into 3 grades: 0.20–0.40 is fair, 0.40–0.75 is good, and ≥ 0.75 is very good [9].

### Statistical methods

The ICC was calculated using SPSS 25.0 statistical software, and the intraobserver and interobserver repeatability was analyzed. At the same time, the Bland–Altman diagram was used to reanalyze the repeatability and consistency. The difference was considered statistically significant at *P* < 0.05.

## Results

The participants were 58 healthy volunteers, including 38 males and 20 females. Age 25–32 years [mean 28 ± 2.2 years]; weight 46–75 kg [mean, 60 ± 12.2 kg]; height 158–176 cm [mean, 167 ± 6.3 cm]; and BMI 19.21–24.2 [mean, 21.3 ± 2.9].

Table 1 shows that the intraobserver reproducibility of contact SWE was good, with an ICC of 0.633 (95% CI 0.380–0.783), and the interobserver reproducibility of contact SWE was good, with an ICC of 0.552 (95% CI 0.243–0.735).

**Table 1:** Repeatability of median nerve elasticity measurements by contact ultrasonography (*n* = 58)

Parameter	Operator 1 (1st)	Operator 1 (2nd)	Operator 2 (1st)	Intraobserver ICC (95% CI)	Interobserver ICC (95% CI)
Emean (Kpa)	29.35 ± 6.67	30.27 ± 6.59	30.73 ± 6.19	0.633 (0.380–0.783)	0.552 (0.243–0.735)

*ICC*—intraclass correlation coefficient, *CI*—confidence interval

Table 2 shows that the intraobserver reproducibility of immersion SWE was very good, with an ICC of 0.975 (95% CI 0.958–0.985), and the interobserver reproducibility of immersion SWE was very good, with an ICC of 0.942 (95% CI 0.902–0.966).

**Table 2:** Repeatability of median nerve elasticity measurements by immersion ultrasonography (*n* = 58)

Parameter	Operator 1 (1st)	Operator 1 (2nd)	Operator 2 (1st)	Intraobserver ICC (95% CI)	Interobserver ICC (95% CI)
Emean (Kpa)	21.54 ± 3.62	22.63 ± 3.66	21.73 ± 3.47	0.975 (0.958–0.985)	0.942 (0.902–0.966)

*ICC*—intraclass correlation coefficient, *CI*—confidence interval

**Fig. 3:**
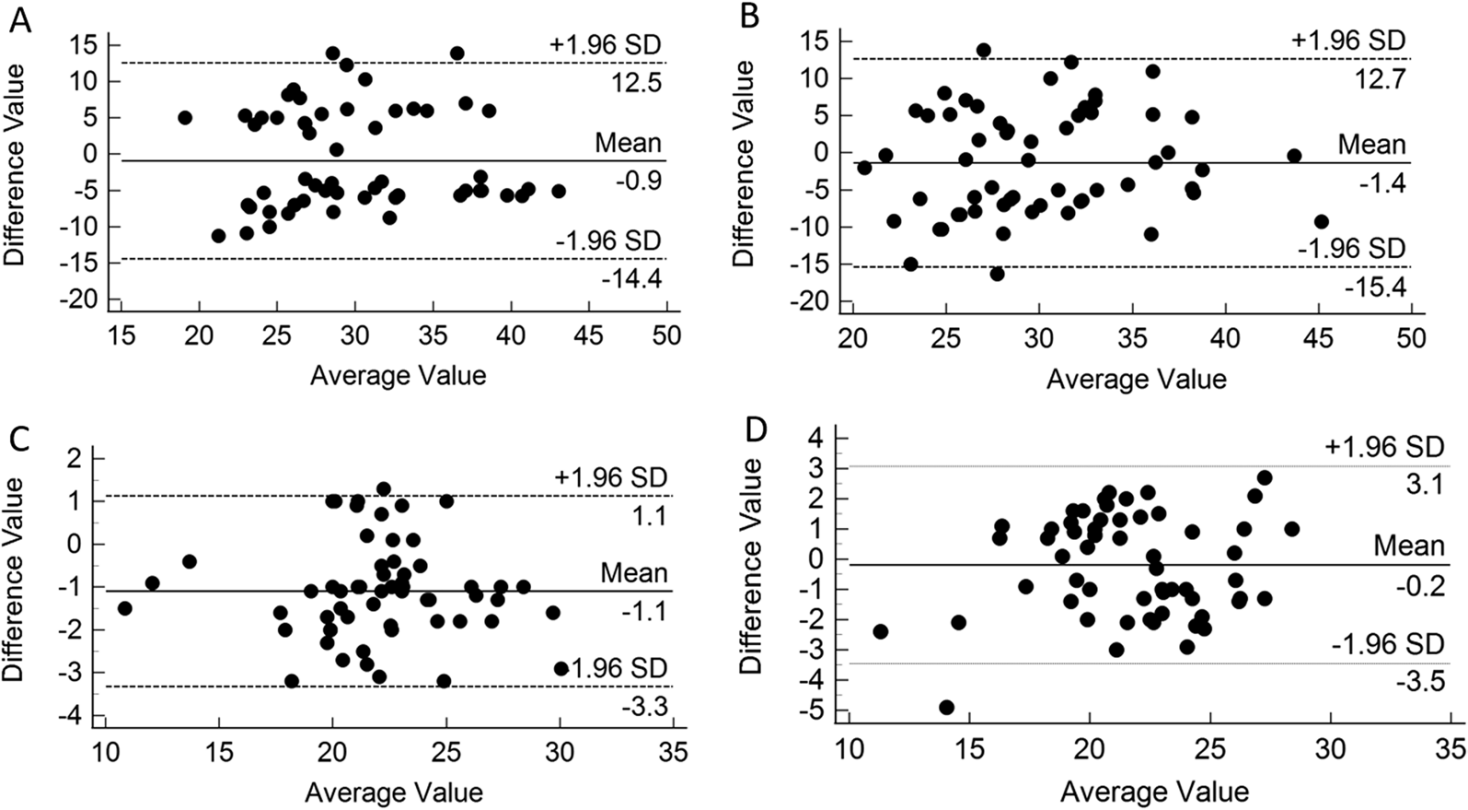
Bland–Altman plot of median nerve elasticity measured by inexperienced operators using two separate methods. **A** Intraobserver Bland–Altman plot by contact ultrasonography. **B** Interobserver Bland–Altman plot by contact ultrasonography. **C** Intraobserver Bland–Altman plot by immersion ultrasonography. **D** Interobserver Bland–Altman plot by immersion ultrasonography

The intraobserver and interobserver Bland–Altman diagram of median nerve elasticity measured by immersion SWE showed that 98% of the points fell within the 95% limits of agreement (LOA). The intraobserver and interobserver Bland–Altman diagram of median nerve elasticity measured by contact SWE showed that 94% of the points fell within the 95% LOA (Fig. 3).

## Discussion

The basic principle of SWE is that sound waves are generated by a sound source, and the sound waves enter the tissue to cause lateral movement of particles, which will generate shear waves. The differences in shear wave propagation velocities in different tissues are used to quantitatively distinguish the properties of tissues [1]. Therefore, no external pressure is required to perform SWE measurements. In contrast, if pressure is applied to the tissue, the accuracy of the measurement will be affected. Some studies have shown that the degree of probe precompression may directly lead to artificial hardening of the measured target, resulting in different measurement results [10]. It has been shown that the degree of precompression influences the measurement of thyroid stiffness [11].

When measuring the elasticity of the median nerve with contact SWE, the ultrasound probe was in direct contact with the skin. Although it is emphasized in clinical work to avoid putting pressure on the skin, to obtain a clear ultrasound image, the probe surface needs to be in full contact with the skin. This unavoidably puts pressure on the skin of the wrist by the ultrasound probe, possible resulting in changes in tissue pre-stiffness, which ultimately affects the accuracy and repeatability of measurements. Moreover, different operators and the same operator will also affect the measurement results because of different operating habits and inconsistent force control. It has been shown that the lack of quantitative indicators of pressure of the operator using the probe can lead to differences in measured results [12].

In addition, because the median nerve is superficial, it is more susceptible to external compression, which reduces accuracy and repeatability of hardness measurement. Although it is clinically recommended to apply a thick layer of couplant to suspend the probe in the couplant for SWE measurement of the median nerve to avoid tissue compression, prolonged suspension of the probe can easily lead to fatigue of the operator's arm and poor image stability. In practice, an ultrasonic coupling pad is also used to measure the SWE of the median nerve, but the compression of the tissue by the weight of the ultrasonic coupling pad cannot be avoided. Therefore, all the above methods will affect the accuracy and repeatability of the final measured value.

When using immersion SWE to measure the median nerve, the liquid is used as the ultrasound medium, and the probe does not directly contact the skin. It avoids the pressure of the probe on the tissue and eliminates the influence of the artificial dependence factor of different operators' habits and strengths. It reduces the influence of external factors that may affect the repeatability of the measurement and improves the accuracy and repeatability of the measurement. The impact of liquid pressure on the skin is minimal, and at the same time, it can fully couple the gap between the skin and the probe so that the tissue can be displayed in a wider range, and it is more convenient for the probe to move and scan.

Contact SWE imaging requires a certain level of skill, thus experienced operators will obtain more accurate results. Therefore, it is recommended that operators undergo corresponding training and perform certain exercises before performing SWE examination. For example, before using contact SWE, operators must practice arm stability to avoid pressure caused by the probe. At the same time, to ensure the quality of the image, the thickness of the applied coupler needs to be evaluated so that the probe is in full contact with the skin. This study shows that doctors without actual SWE operation experience can also obtain good repeatability for median nerve measurement using immersion SWE, indicating that immersion SWE does not require a certain level of skill in performing measurements.

Immersion SWE also has certain limitations and is not suitable for patients with skin damage, ulcers, or inflammation. In addition, the subjects of this study were healthy adults, so whether immersion SWE has the same high reproducibility in patients with median neuropathy caused by diabetes, carpal tunnel syndrome and other diseases needs further research to be confirmed. Whether immersion ultrasound can improve the diagnostic efficiency for CTS still needs further study, which is the direction of our future research. The experience of the operator will affect the repeatability of the measurement [13, 14]; thus, comparisons between inexperienced operators and ultrasound experts in the use of immersion SWE are also needed. At the same time, the measured object in this study was the right median nerve, and whether there is a difference in measurements of the median nerve between the left and right sides needs to be further verified.

In conclusion, we believe that the main reasons why immersion SWE improves the repeatability of median nerve measurement by doctors without SWE operation experience are as follows: 1. The liquid medium avoids the pressure effect of the probe on the skin. 2. The liquid acts as a medium so that the probe is not in direct contact with the skin, which avoids the influence of operator experience. In conclusion, we believe that immersion ultrasound can improve the reproducibility of median nerve elasticity measurement by doctors without SWE operation experience, and it is worthy of clinical promotion.

## Data Availability

Data may be obtained from the corresponding author upon reasonable request.

## References

[1] Nattabi HA, Sharif NM, Yahya N, Ahmad R, Mohamad M, Zaki FM, Yusoff AN (2022). Is diagnostic performance of quantitative 2D-shear wave elastography optimal for clinical classification of benign and malignant thyroid nodules? A systematic review and meta-analysis. Acad Radiol.

[2] Tian J, Liu Q, Wang X (2017). Application of 3D and 2D quantitative shear wave elastography (SWE) to differentiate between benign and malignant breast masses. Sci Rep.

[3] Dikici AS, Ustabasioglu FE, Delil S, Nalbantoglu M, Korkmaz B, Bakan S, Kula O, Uzun N, Mihmanli I, Kantarci F (2017). Evaluation of the tibial nerve with shear-wave elastography: a potential sonographic method for the diagnosis of diabetic peripheral neuropathy. Radiology.

[4] Ghajarzadeh M, Dadgostar M, Sarraf P, Emami-Razavi SZ, Miri S, Malek M (2015). Application of ultrasound elastography for determining carpal tunnel syndrome severity. Jpn J Radiol.

[5] Kantarci F, Ustabasioglu FE, Delil S, Olgun DC, Korkmazer B, Dikici AS, Tutar O, Nalbantoglu M, Uzun N, Mihmanli I (2014). Median nerve stiffness measurement by shear wave elastography: a potential sonographic method in the diagnosis of carpal tunnel syndrome. Eur Radiol.

[6] Cingoz M, Kandemirli SG, Alis DC, Samanci C, Kandemirli GC, Adatepe NU (2018). Evaluation of median nerve by shear wave elastography and diffusion tensor imaging in carpal tunnel syndrome. Eur J Radiol.

[7] Lin CP, Chen IJ, Chang KV, Wu WT, Özçakar L (2019). Utility of ultrasound elastography in evaluation of carpal tunnel syndrome: a systematic review and meta-analysis. Ultrasound Med Biol.

[8] Moran L, Royuela A, de Vargas AP, Lopez A, Cepeda Y, Martinelli G (2020). Carpal tunnel syndrome: diagnostic usefulness of ultrasound measurement of the median nerve area and quantitative elastographic measurement of the median nerve stiffness. J Ultrasound Med.

[9] Benchoufi M (2020). Interobserver agreement issues in radiology. Diagn Interv Imaging.

[10] Liu B, Liang J, Zheng Y (2015). Two-dimensional shear wave elastography as promising diagnostic tool for predicting malignant thyroid nodules: a prospective single-centre experience. Eur Radiol.

[11] Lyshchik A, Higashi T, Asato R (2005). Elastic moduli of thyroid tissues under compression. Ultrason Imaging.

[12] Adler DD, Carson PL, Rubin JM (1990). Doppler ultrasound color flow imaging in the study of breast cancer: preliminary findings. Ultrasound Med Biol.

[13] Tekin L, Kara M, Türker T, Ozçakar L (2011). Shoulder measurements in the early period of ultrasound learning: chasing the butterfly?. J Rehabil Med.

[14] Özçakar L, Kara M, Tekin L, Karanfil Y, Esen E, Utku B, Güven SC, Çağlayan G, Youssefi A, Pitruzzella M, Ciocchetti E, Açikel C (2013). Effect of supervision on ultrasonographic measurements. A blinded randomized cross-over study. Eur J Phys Rehabil Med.

